# Automated in vivo enzyme engineering accelerates biocatalyst optimization

**DOI:** 10.1038/s41467-024-46574-4

**Published:** 2024-04-24

**Authors:** Enrico Orsi, Lennart Schada von Borzyskowski, Stephan Noack, Pablo I. Nikel, Steffen N. Lindner

**Affiliations:** 1 The Novo Nordisk Foundation Center for Biosustainability, Technical University of Denmark, 2800 Kongens Lyngby, Denmark; 2 Institute of Biology Leiden, Leiden University, 2333 BE Leiden, The Netherlands; 3 Institute of Bio- and Geosciences, IBG-1: Biotechnology, Forschungszentrum Jülich, 52425 Jülich, Germany; 4 Max Planck Institute of Molecular Plant Physiology, 14476 Potsdam-Golm, Germany; 5 Department of Biochemistry, Charité Universitätsmedizin Berlin, corporate member of Freie Universität Berlin and Humboldt-Universität, 10117 Berlin, Germany

**Keywords:** Synthetic biology, Biocatalysis

## Abstract

Achieving cost-competitive bio-based processes requires development of stable and selective biocatalysts. Their realization through in vitro enzyme characterization and engineering is mostly low throughput and labor-intensive. Therefore, strategies for increasing throughput while diminishing manual labor are gaining momentum, such as in vivo screening and evolution campaigns. Computational tools like machine learning further support enzyme engineering efforts by widening the explorable design space. Here, we propose an integrated solution to enzyme engineering challenges whereby ML-guided, automated workflows (including library generation, implementation of hypermutation systems, adapted laboratory evolution, and in vivo growth-coupled selection) could be realized to accelerate pipelines towards superior biocatalysts.

## Abstract (web summary)

Achieving cost-competitive bio-based processes requires development of stable and selective biocatalysts. In this Perspective, the authors propose an integrated solution combining growth-coupled selection with machine learning and automated workflows to accelerate development pipelines.

## Introduction

The development of tailored and efficient bio-based processes is essential for applications as diverse as biopharmaceutical production, industrial biotechnology, food technology, crop improvement, and bioremediation. To establish such profitable bio-based processes, biocatalysts that can perform substrate-to-product conversions with high volumetric productivities (g_product_ L^−1^ h^−1^), yields (g_product_ g_substrate_^−1^), and selectivities (enantiomeric excess) are essential^1^. To reach improvements in these performance indicators and optimize chemical conversions, enzyme engineering has been developed as one of the pillars of synthetic biology^2^, realizing enzyme optimization and development from the single reaction step to entire metabolic pathways^2,3^.

Current efforts in bioengineering aim at designing biological systems that provide enzymatic activities beyond what has been developed and optimized by nature^2,4,5^. Implementing these innovations can further develop a bio-based economy^6–8^. Hence, it is desirable to design novel, new-to-nature enzymatic activities as the key parts needed to assemble complete synthetic pathways^2,9^ or used in enzyme-driven catalysis applications directly in synthetic processes (e.g., in the striking case of in vitro conversion of CO_2_/H_2_ or methanol into starch^10^). However, creating and optimizing such new-to-nature reactions is a challenging task, for which the use of rational protein design accompanied by in vitro enzyme activity measurements or adaptive laboratory evolution (ALE) might not be sufficient^11,12^.

At this point, directed evolution comes in handy, as it allows to perform Darwinian evolution in a test tube by increasing mutation and recombination rates within a target gene^13,14^. Two types of directed evolution approaches are possible and differ in the environment where the evolution takes place. In vitro-directed evolution occurs outside a living organism, whereas in vivo evolution takes place within living systems. Both strategies have pros- and cons- which have been discussed elsewhere^15,16^. In recent years, in vivo-directed evolution approaches have emerged as promising tools to use in protein engineering campaigns^11,16^. The use of these approaches combined with growth-coupled selection (meaning coupling the enzymatic activity of interest to microbial fitness) has been applied for different optimization strategies^17,18^. At the same time, automated biofoundries are becoming pivotal in supporting high-throughput efforts for engineering biology^19–22^. Hence, the use of these infrastructures for protein engineering is gaining momentum^23^. Moreover, the use of artificial intelligence (AI) and machine learning (ML) is aiding important endeavors in the design of new biological systems, from protein- to organism level^24–27^. Therefore, we are witnessing a paradigm shift in our ability and capacity to engineer biological systems. A combination of these technologies might result in the establishment of self-driving labs and workflows, which potentially accelerate scientific discoveries and innovation while reducing human errors^28–30^. In this article, we discuss how the integration of ML, in vivo continuous evolution, and the use of automated biofoundries will accelerate the generation of new and competitive biocatalysts capable of supporting the transition towards a circular bio-based economy. Definitions of the most important technical terms used within the text are described in the Box 1.

### An integrated workflow for accelerating in vivo enzyme engineering

**Fig. 1:**
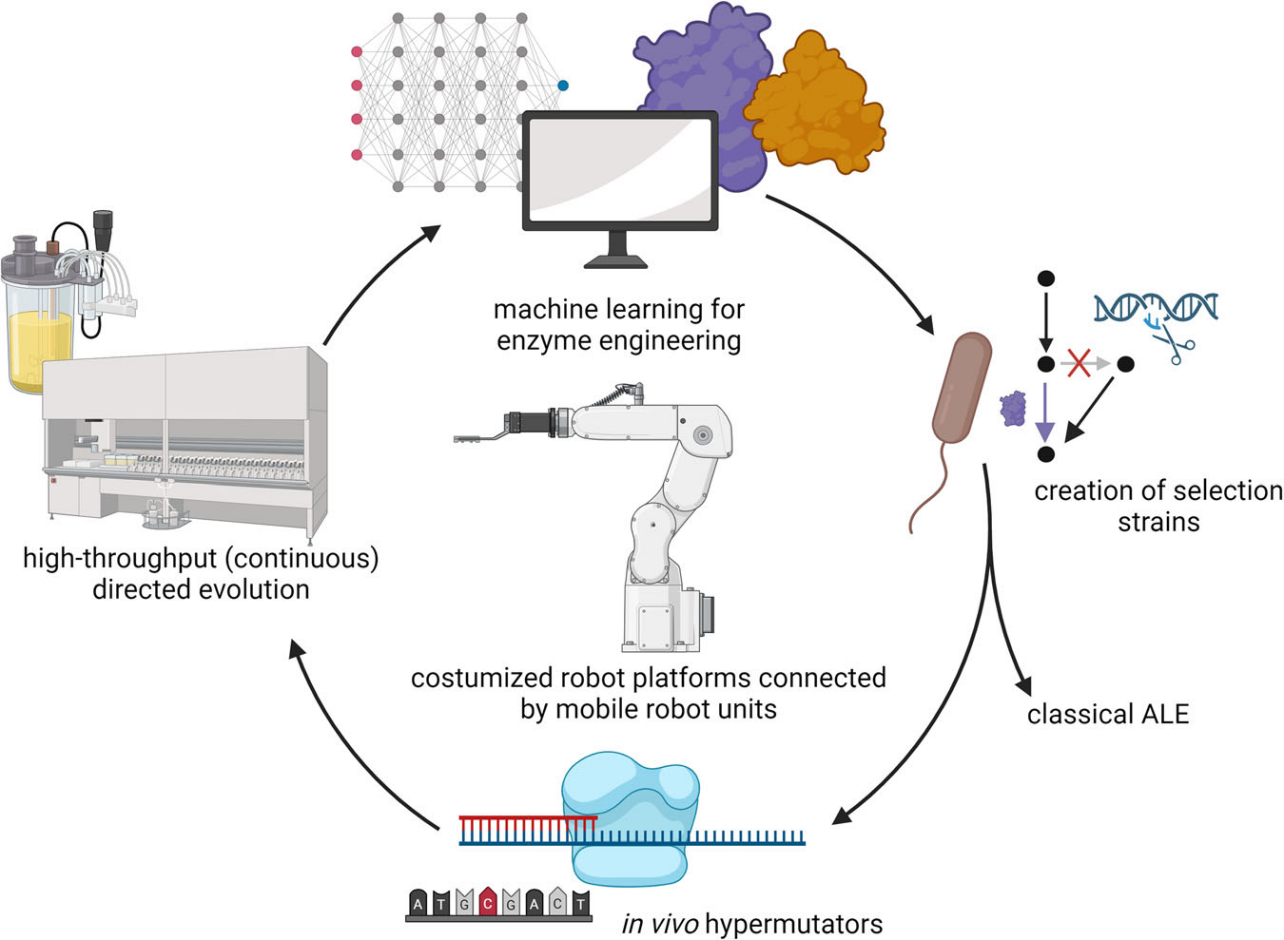
Conceptual overview of the pipeline proposed in this article. This workflow aims at generating superior catalysts by combining the use of machine learning, growth-coupled selection, in vivo hypermutators, and high-throughput cultivations. Each part can be envisioned as a stand-alone module, which can, in principle, be connected using mobile robot units. Created with BioRender.com.

To enable the workflow proposed in this article, different fields of expertize need to be integrated (Fig. 1). In brief, ML is used as input for (i) predicting the modifications required for engineering the target enzyme(s) and (ii) supporting the design of auxotroph selection strains by suggesting target genes to delete. Then, these selection strains are created through gene deletions. Subsequently, in vivo hypermutators can be exploited to increase the mutation rate within the target gene. Following the principle of growth-coupled selection, high-throughput and continuous cultivation platforms can be used for enriching the microbial population with clones containing the evolved target enzyme(s). Finally, sequencing of the enriched clones can inform the success of the evolution campaign. This step can also benefit from the use of ML-guided computational tools. If necessary, this pipeline can be iterated through several rounds.

The abovementioned steps should be intended as stand-alone workflows, which can be integrated by mobile robot units (Fig. 1). In the following sections of the manuscript, we dive more in-depth into the different aspects of this pipeline, eventually suggesting their integration using state-of-the-art automated workflows. Finally, we discuss caveats and limitations of this concept.

### Enzyme engineering generates improved biocatalysts

Mastering enzyme engineering is vital to enable the optimization of existing bioprocesses or the exploration of new ones. Here, we cannot give a comprehensive overview of this large and rapidly developing area of research, but rather present key approaches and concepts. For a more detailed summary, the reader is referred to the existing literature^4,31–33^.

The targets for enzyme engineering are highly diverse. To broaden the substrate range, the active site must be opened and remodeled; to improve the substrate specificity or enantioselectivity, the active site must be altered to only accommodate one type of desired substrate or intermediate^33^; to develop novel catalytic functions, general principles of catalysis and transition state stabilization have to be applied to modify a suitable scaffold enzyme. To increase the thermal stability of enzymes and allow the catalysis of industrial processes at high temperatures, enzymes must be modified by introducing additional hydrogen bonds and salt bridges, rigidizing flexible residues, creating a more compact core region, or decreasing surface area hydrophobicity^34,35^. Computational tools and predictions can help in identifying relevant amino acid residues and regions of interest to be mutated^36,37^. Prediction of relevant residues to be mutated can be supported by experimentally determined protein structures, or—in their absence—by the data-driven protein structure prediction tool AlphaFold^38^, which has made high-quality protein structure models easily available for the global research community since 2021.

**Fig. 2:**
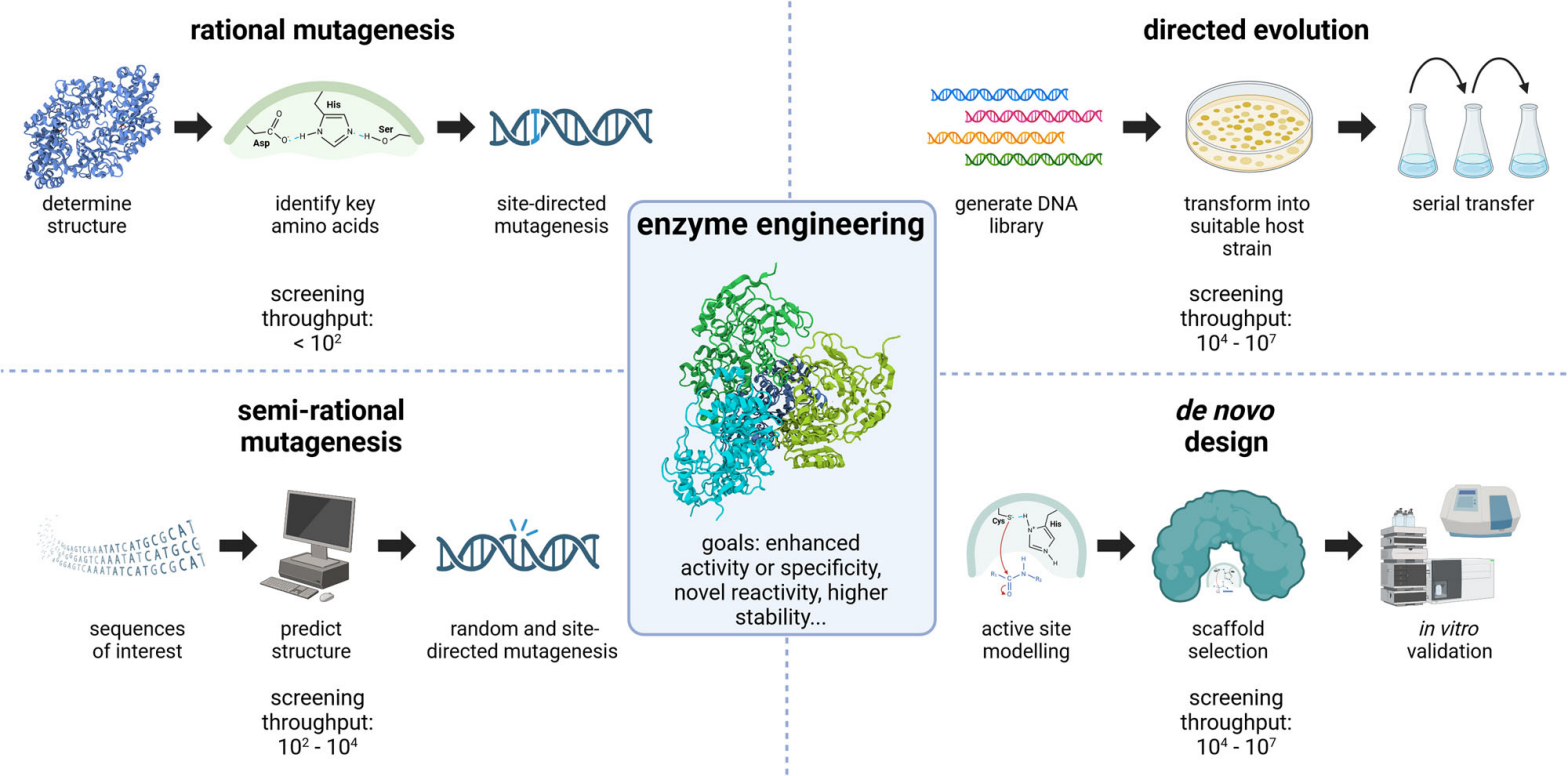
Enzyme engineering is applied to improve the properties of biocatalysts in a desired way. This includes increasing enzyme activity, substrate specificity, or enantioselectivity, introducing novel reactivities, or improving protein stability, among other goals. Methods that are applied for this purpose range from rational mutagenesis of key amino acids to semi-rational approaches and directed evolution of gene sequences from DNA libraries. De novo enzyme design can be applied to generate biocatalysts that are free from the constraints of existing enzymes. Created with BioRender.com.

**Fig. 3:**
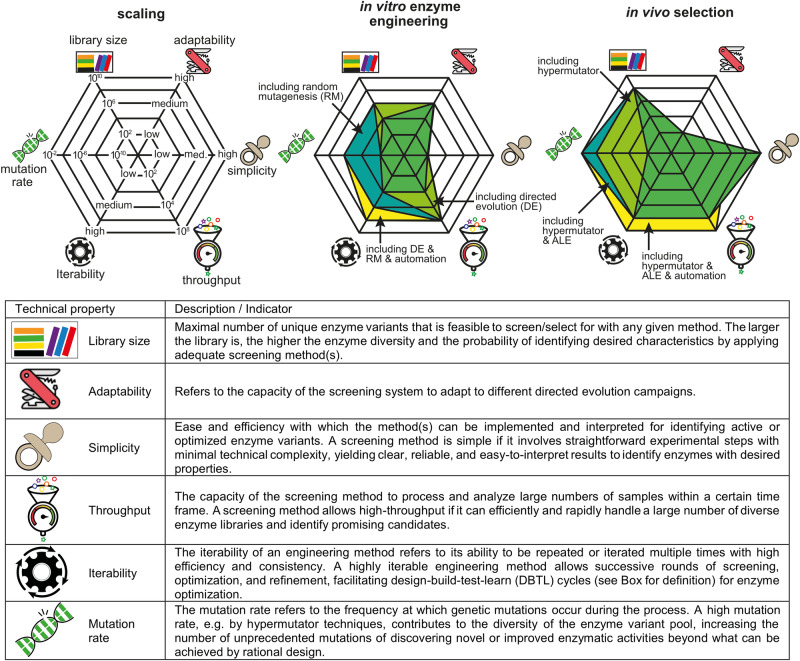
Comparison of in vitro and in vivo approaches for enzyme optimization. The figure shows and scores the properties of the two methods for several key performance indicators (KPIs). Moreover, the use of additional technical improvements to the approaches is included, such as random mutagenesis (RM), directed evolution (DE), hypermutators and adaptive laboratory evolution (ALE). The impact of these improvements on the KPIs is also depicted in the figure.

Previously established techniques of enzyme engineering, such as rational mutagenesis based on in silico predictions or structure analysis, semi-rational mutagenesis (i.e., the combination of site-directed mutagenesis with random mutagenesis or directed evolution^39^), and directed evolution of a suitable parent enzyme^13,40^, can provide the starting points for screenings of improved biocatalysts and iterative cycles of enzyme development (Fig. 2). However, efficiently enhancing catalytic properties of enzymes requires maneuvering through complex and rugged fitness landscapes, where the relationship between enzyme sequence (genotype) and functional characteristics (phenotype) is difficult to predict. This means that optimization trajectories frequently result in diminishing returns (i.e., additional mutations only result in minimal improvements of an enzyme) and undesired tradeoff effects (e.g., substrate specificity is improved, but enzyme turnover number is strongly decreased)^41,42^. When the abovementioned methods no longer yield enhancements, it often remains uncertain if an enzyme has already reached its maximum catalytic efficiency, or if there are other possible combinations of mutations that could generate further improvements^43^. Therefore, the construction of large combinatorial enzyme libraries is a key approach in enzyme engineering (Fig. 2). By introducing diversity into enzyme sequences, libraries can be screened or selected for desired properties. High-throughput screening methods, including droplet-based microfluidics and fluorescence-activated cell sorting (FACS), enable the isolation and identification of enzymes with improved features, as long as a suitable readout is available (Fig. 3).

In addition to the previously discussed methods, ML approaches are now being more commonly employed to identify data patterns that aid in forecasting protein structures, enhancing enzyme stability, solubility, and function, predicting substrate specificity, and facilitating rational de novo protein design^27,44,45^.

### De novo enzyme design complements enzyme engineering

The aim of de novo enzyme design is the development of novel enzymes with desired functions from scratch, without relying on naturally occurring enzymes as starting points. Various protein design algorithms, such as Rosetta^46,47^ (https://www.rosettacommons.org/), have been developed to predict and optimize enzyme sequences based on desired functions. Rosetta relies on the mechanistic modelling of proteins using energy fields to guide the design process and explore the vast sequence space for enzyme engineering^31^. Already 15 years ago, the first high-profile designer enzymes, e.g., for the catalysis of retro-aldol reactions, were reported^48^. Some current highlights in this rapidly growing field include the de novo design of an eight stranded β-barrel protein that functions as a retro-aldolase, whose activity and stereoselectivity were further improved using directed evolution^49^, and the creation of artificial luciferase enzymes from scratch, whose catalytic efficiency is comparable to that of natural luciferases, while having a much higher substrate specificity and very high thermostability^50^. The de novo design of enzymes that bind complex cofactors, such as heme irons, is also not an obstacle anymore: recently, the creation of a heme enzyme with a tunable substrate-binding pocket and its further engineering into an efficient carbene transferase was reported^51^. The combination of protein design with iterative mutagenesis for efficient enzyme engineering should not be underestimated. A good example is the conversion of a designed enzyme with modest activity for carbon-carbon bond formation between aldehydes and enones^52^ into a highly efficient biocatalyst via fourteen rounds of both local and global mutagenesis, coupled to high-throughput spectrophotometric assays as time-efficient readout^53^.

It seems likely that de novo protein design will be feasible and widely used for all types of enzymes in the near future. The fine-tuning of the deep learning neural network RoseTTAFold^54^ on protein structure denoising tasks resulted in RFdiffusion, a generative model of protein backbones with outstanding performance on protein monomer design, enzyme active site scaffolding (Fig. 2), and metal-binding protein design, which only requires simple molecular specifications as input^55^.

Computational enzyme design is especially valuable to realize a novel metabolic pathway in which a natural enzyme for one reaction step is lacking^56^. Here, de novo design can supply enzyme candidates that catalyze the desired conversion, often only with initially low activities. These enzymes can subsequently be improved by mutagenesis and directed evolution, making it possible to implement efficient new-to-nature bioconversion routes.

### ML-supported pathway design increases the engineering design space

The design and implementation of non-natural metabolic pathways is a complex and highly time-consuming task. ML can alleviate this challenge by automating several stages of the pathway design process^24^. Specifically, ML algorithms can efficiently predict and analyze metabolic reactions, aiding in retrobiosynthesis approaches (i.e., the identification of potential pathways for the production of specific, desired compounds^57–59^). Furthermore, ML is a powerful tool that can efficiently detect patterns in large sets of data. It has been extensively employed for analyzing datasets obtained through high-throughput technologies in order to create data-based models for intricate bioprocesses. The integration of ML with the Design-Build-Test-Learn cycle commonly applied in synthetic biology can accelerate the development process^26^. It can also assist in optimizing the metabolic engineering process, by intelligently exploring and designing different combinations of enzymes and genetic modifications to enhance pathway efficiency and yield.

A good example of this is METIS, a flexible active ML workflow that enables the efficient optimization of biological targets with minimal experiments^60^. The effectiveness of this approach was demonstrated across a range of applications, such as cell-free transcription and translation, genetic circuits, and a synthetic carbon dioxide fixation cycle with 27 variables. The performance of these systems was enhanced by one to two orders of magnitude. Moreover, the workflow identified the relative importance of individual factors in system performance, uncovering previously unknown interactions and bottlenecks. It can be expected that similar workflows will realize the easy optimization and prototyping of diverse genetic and metabolic networks by a broad user base in the near future.

Since characterization, structure prediction, and de novo design of enzyme function as well as drafting and prototyping of metabolic pathways, largely benefit from the plethora of innovative methods summarized above, the possible design space of biological engineers vastly increases in size. Therefore, in vitro testing and screening of enzymes and metabolic pathways might be limited in capacity with consequent constraints in the optimization process of engineered biological systems (Fig. 3).

### Selection strains allow high-throughput in vivo enzyme screening

As discussed above, an alternative to in vitro testing for enzyme or pathway screening is represented by in vivo assessment using auxotroph sensor strains (henceforth referred to as selection strains), systematic growth-coupling designed by modeling^61^, or using antimetabolite selection strains^62^. These rely on the selective pressure generated by metabolite analogs which inhibit growth. As a consequence, growth restoration is possible via enhanced enzyme production or synthesis of the target molecules^62^. However, it is important to note at this stage that in vivo selection might not be possible to exploit if the enzyme to be optimized cannot be linked to the metabolism of the host cell in a suitable way to enable growth-coupling, or when it does not produce an antimetabolite.

In the context of this article, we focus on auxotroph selection strains as a platform for enabling in vivo enzyme screening and evolution. In general terms, these selection strains are obtained through gene deletions which interrupt the host’s metabolic network. In other words, in such strains, the biosynthesis of key biomass precursors or essential metabolic functions is blocked^63,64^. Growth of these strains can be restored when supplying the “missing” biomass building blocks or when introducing metabolic modules (i.e., enzymatic reactions of interest) that reestablish the biosynthesis of essential metabolites. Hence, growth becomes a straightforward readout of the module’s activity^63,64^. Multiple selection strains can be generated for the same auxotrophy so that such a demand can cover different ranges of sensitivity (i.e., pulling force of the selection)^65^. This feature exhibits the advantage of creating different intensities of selective pressure, which can be exploited for screening purposes. In other words, selection strains are convenient platforms to explore for enzyme evolution purposes^17,18^, and their throughput is limited only by the transformation efficiency^66,67^.

**Fig. 4:**
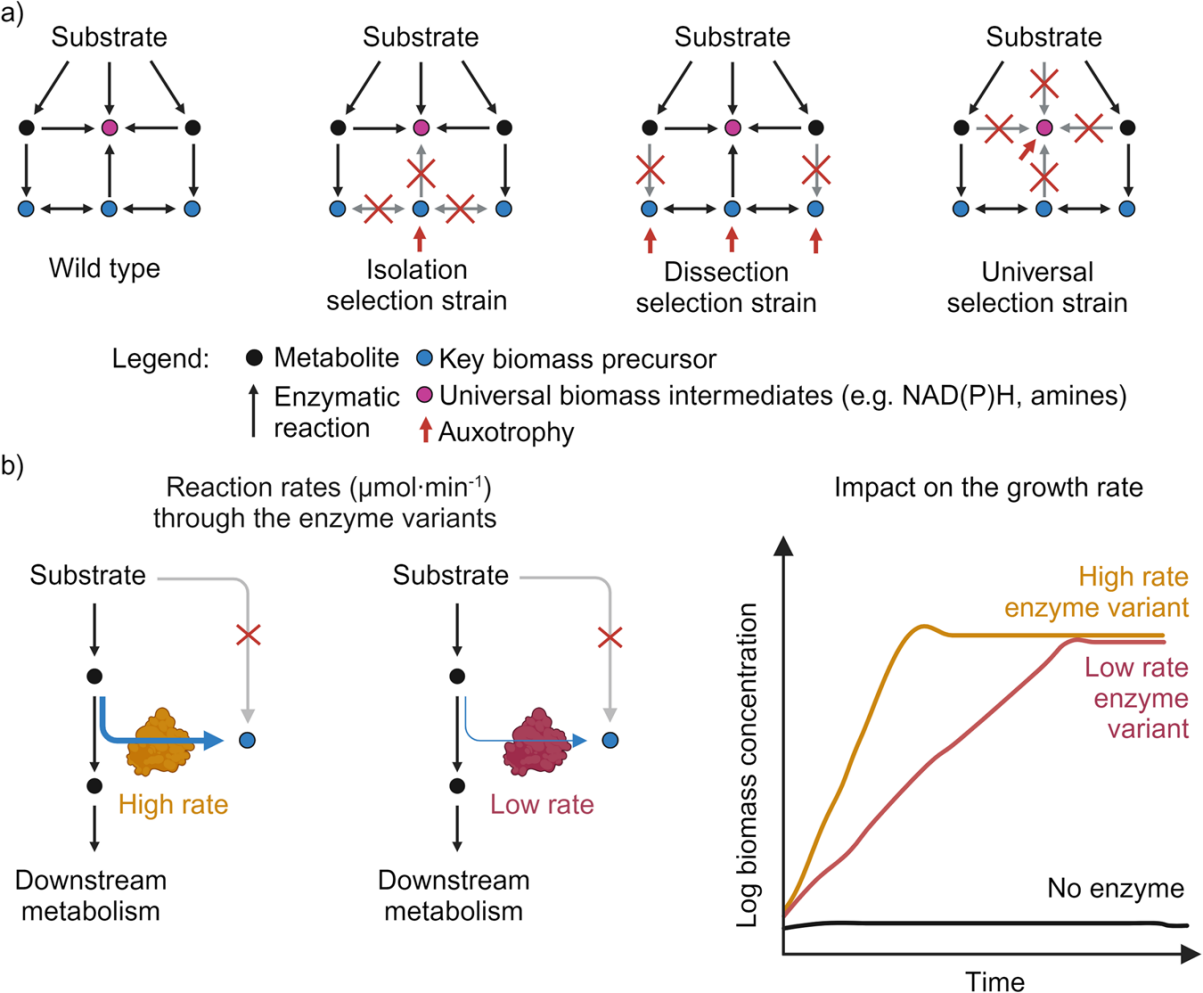
Adopting dedicated selection strains supports efficient enzyme screening. **a** Different types of auxotrophic selection strains can be used for in vivo enzyme evolution. The first group includes isolation and dissection strains; both strains are incapable of synthesizing essential biomass precursor(s) by blocking or isolating specific metabolic nodes. The second group is represented by strains unable to produce universal biomass intermediates (e.g., synthesis of NAD(P)H or amines). Red arrows indicate the auxotrophy generated. **b** Effect of reaction rates on selection strain’s growth profile. A selection strain produces two different enzyme variants capable of replenishing synthesis of a key biomass precursor through their enzymatic activity. The two enzyme variants display different reaction rates (µmol min^−1^). These different reaction rates will impact the in vivo selection, as the two selection strains can be distinguished by their different growth rates (h^−1^). Created with BioRender.com.

Selection strains can be categorized into two main groups, depending on how the auxotrophy is designed (Fig. 4a): to the first group belongs to strains presenting metabolic “isolation” or “dissection”, whereas the second one includes strains deficient in a universal metabolic task (i.e., cofactor regeneration or provision of amino groups). The first group includes strains that cannot generate an essential biomass precursor molecule or an intermediate metabolite responsible for the synthesis of a biomass precursor molecule (isolation strains). This type of strain was crucial for, e.g., the stepwise implementation of the different modules of the reductive glycine pathway prior to the demonstration of full formatotrophy^68,69^. Similarly, “dissection” strains are incapable of synthesizing a key biomass component or one of its precursors. Moreover, in this case, the segmentation of the metabolic network is not limited to a single key metabolite but rather to a whole metabolic region (including several biomass precursor molecules). Such a broader selection range requires a higher enzymatic activity to support growth. Several studies are based on the use of dissection strains, and include, e.g., the generation of a hemi-autotrophic and an autotrophic *E. coli* growing through the Calvin-Benson-Bassham cycle^70,71^, full formatotrophic growth via the reductive glycine pathway^72^, test of shunts for the ribulose monophosphate^73^ or the Gnd–Entner–Doudoroff^74^ cycles. Another striking example of this type of selection strains is a 3-phosphoglycerate sensor that can respond to several orders of magnitude of 3-phosphoglycerate concentrations^65^.

The second group of selection strains includes mutants unable to perform a metabolic function common to multiple biochemical blocks (Fig. 4a). Examples of this sort are mutants deficient in cofactor regeneration, either in the form of NADH^75^ or NADPH^76^. A plethora of growth-coupled selection strategies have been developed using this type of auxotrophy, both for enzyme screening and for directed evolution campaigns at different throughput levels^66,77–83^. Strains that lack the ability to fix ammonium to make amino acids and other essential amine metabolites also belong to this category. These can be used to select for a broad range of amine-generating reactions, e.g., for the exploration of alternative amination routes^84^ or for supporting the directed evolution of amine-related enzymes^85^.

Once the selection strains are equipped with the module of interest, growth restoration works as a proxy for the module’s enzymatic activity^63,64^. In particular, the growth rate µ (h^−1^) can be used as a coarse-grained proxy for the reaction rate (µmolˑmin^−1^) of the target enzyme in in vivo, i.e., in the context of a dedicated selection strain (Fig. 4b). For example, a selection strain expressing two different gene variants encoding for the same enzymatic activity (e.g., unevolved and evolved) might present a different growth rate for the two clones as a consequence of different reaction rates through the target enzymes (Fig. 4b). Moreover, changes in expression levels of the gene of interest (also as a consequence of evolution) might result in an improved growth rate. In summary, using growth-coupled selection strategies represents a cheap and resourceful approach for determining enzymatic activities in vivo.

### Combining growth-coupling to directed evolution for new phenotypes

When exploring new-to-nature enzymatic reactions (e.g., formyl-phosphate reductase^86^ or glycolyl-CoA carboxylase^87^), it can be useful to expand the solution space of mutations which can be screened. In this situation, directed evolution becomes a useful tool as it increases genetic diversity within a sequence of interest, provided that a high-throughput per experiment can be achieved. Several directed evolution strategies have been developed through the years. They are divided mainly into two groups, based on where the diversification of the starting genetic sequence occurs: in vitro and in vivo mutagenesis. Both approaches have been extensively reviewed in literature^11,13,14,16,17,23,40,67,88,89^.

In vitro mutagenesis approaches generate a library of gene variants in a test tube, which is then transformed into an adequate strain and screened for a readout of interest. Hence, transformation efficiency becomes the bottleneck for the number of gene variants one could recover and screen. In aid to this limitation, microfluidics solutions for high-throughput electroporation are becoming available which circumvent these shortcomings^90^. The most common in vitro techniques include (but are not limited to) error-prone PCR, site saturation mutagenesis and recombination-based DNA shuffling. In vitro-directed evolution can also be combined with the use of selection strains for in vivo screening of the evolved enzyme activity, as in the case of a formate dehydrogenase with improved specificity toward NADP^+^ ^80^. We refer to excellent reviews on the topic for more in-depth comparisons of the in vitro techniques available^13,14,23^.

The use of in vivo mutagenesis strategies allows to bypass the bottleneck of transformation efficiency and perform gene diversification within the cell. Multiplex automated genome engineering (MAGE) as well as CRISPR-Cas technologies^91^ or zinc finger nucleases^92^ mediated tools are examples of in vivo directed mutagenesis based on mediated allelic replacement^93^. In the case of MAGE, a pool of single-stranded DNA oligos with degenerated sequences is transformed into cells, which generates a variety of genetic modifications in vivo. By iterating this transformation step, it is possible to enhance library complexity and generate a pool of mutants which can be screened once plated on e.g., selective agar plates^93^. The use of the abovementioned methods has been extended to multiple species beyond model laboratory strains^94^. Altogether, the creation of genome-edited library strains instead of plasmid-based ones enables rapid adjustment of the strategy depending on the results of the preceding iteration.

An additional benefit of in vivo enzyme library generation is the ability to combine library generation with techniques that significantly elevate the mutation rate of the target gene. When employing selection strains for enzyme development, mutagenesis takes place concurrently with the selection of the desired phenotypic trait. These hypermutation methods facilitate rapid introduction of mutations into a gene, increasing the mutation rate (naturally between 10^−10^ and 10^−9^ to as high as 10^−4^)^11^. Thus, these methods surpass the typical mutation rates achieved through ALE experiments, enabling the quick generation of diversified enzyme variants. Moreover, they significantly reduce the mutation or activation of off-target enzymes that might circumvent selection in the chosen strain, e.g., by activating silent genes or by mutating an enzyme to enhance its promiscuous activity. Hence, these techniques facilitate a more thorough exploration of the fitness landscape, aiding in the creation of enzyme variants that surpass local fitness maxima.

Several hypermutation techniques have been developed, as extensively reviewed recently^11^. Most of these methods are based on error-prone DNA polymerase (OrthoRep)^95,96^, nCas9-mediated DNA nicking combined with error-prone DNA polymerase (EvolvR)^97^, or nucleobase deaminase/T7 RNA polymerase (MutaT7)^98^. These techniques have been continually refined since their inception, with ongoing development focused on enhancing mutation rates and profiles. Derivatives of MutaT7 technology include e.g., extension of this technology to *S. cerevisiae*^99^, improvement of its mutation rate^100^, and fusion of a new deaminase combined with the introduction of dCas9 to obtain more control over T7 RNA polymerase^101^. Further utilization of OrthoRep allowed, e.g., to evolve custom antibodies to display on yeast’s surface^102^ or an improved version of tryptophan synthase for synthesizing L-tryptophan from indole and L-serine^103^. Another recent update of the OrthoRep system claims an improved rate of in vivo substitution per base (>10^−4^)^104^. Also, during the revision of this manuscript, a new technique was published that relies on an orthogonal DNA polymerase^105^. In this system, user-defined DNA is introduced into an *E. coli* cell in such a way that it is selectively copied and mutated by a distinct replication machinery which is independent from the one responsible of duplicating the strain’s genome. This approach resulted in the enhancement of the mutation rate in the target replicon between two to four orders of magnitude^105^.

In addition to enzyme-based hypermutator tools, phages have been utilized as vectors to introduce variations into a target gene. This approach, known as phage-assisted continuous evolution (PACE), has caught significant interest^106^. In PACE, engineered phages are employed to introduce sequence variations. Leveraging the remarkably short lifecycles of phages, this method accelerates evolution cycles and enhances mutation rates in a gene of interest in the host bacterium^107^.

In conclusion, state-of-the-art directed evolution techniques are available to develop enzymatic reactions in vivo, simplifying optimization workflows. Additionally, an expansion of the solution space beyond prediction, achievable with targeted hypermutator tools, introduces the necessary genetic diversity. The combination of these techniques^108,109^ maximizes the diversity of the library, with its size theoretically constrained only by the number of cells in the culture. Finally, the combination of the workflow with ALE promotes the enrichment of more optimal variants (Fig. 3).

### ALE further enhances emerging phenotypes

Once the round(s) of directed mutagenesis enable the emergence of the activity of interest, it is possible to exploit the power of ALE to further enhance the target reaction rate. Many excellent reviews discuss the set of techniques associated with this approach, and we refer to them for a more thorough read; see for example^12,110–113^. In the context of enzyme evolution, the use of ALE in combination with selection strains has also been described^114–116^.

To achieve an improved phenotype, 100–500 generations are generally sufficient^112^. These can be obtained using mainly three different experimental approaches: (i) serial batch dilutions or continuous cultivation either as (ii) chemostat or (iii) turbidostat^111^. In a serial batch, a growing microbial population is propagated by serial dilutions over time while the stress factor is kept constant or increased. In this setup, the growth conditions are dynamic throughout the growth, and the moment of growth chosen for dilution has an impact on the phenotype that is being selected for. Instead, in a chemostat, the culture conditions are kept constant throughout the cultivation; influx and efflux of medium are equal, and the dilution rate sets the specific growth rate of the microbial population. A steady exponential growth is imposed on growing cells while a limiting essential nutrient determines the selective pressure. Subpopulations slower at consuming the limiting nutrient will be washed out from the cultivation and removed from the bioreactor. In this cultivation setup, both the concentration of the limiting nutrient in the feeding and the dilution rate can be controlled by the user. An overview on the basis of ALE using chemostats can be found in literature^117,118^. A turbidostat differs from a chemostat as its dilution rate is controlled by the turbidity of the culture. Here, the goal is to maintain the turbidity constant. This system allows to select for a population of cells capable of growing at *µ*_max_ and does not require the introduction of a limiting nutrient. The use of turbidostat in studying enzyme evolution has also been reported in literature^119^. Hence, depending on the phenotype one wants to select for, these different cultivation conditions can be used to support ALE efforts.

One common characteristic of the abovementioned ALE approaches is the constant selective pressure that is imposed on the system. An emerging alternative consists of the use of oscillating pressures for traversing different fitness landscapes and increasing the chances of reaching a global maximum for the phenotype of interest^120^. In particular, the use of this strategy allows the exploration of mutations that would be otherwise deleterious during constant pressure. This approach allowed e.g., a change in cofactor specificity when an NADPH-auxotrophy was imposed in *E. coli*^116^. Therefore, the use of such oscillation in combination with directed evolution might allow to evolve enzyme activities through changing rugged fitness landscapes^120^. In summary, we posit that ALE should be regarded as a complementary approach supporting directed evolution for the emergence of novel enzymatic reactions in biocatalysts (Fig. 3).

### In the quest for optimal microbial hosts for in vivo enzyme engineering

While *E. coli* and *S. cerevisiae* have been historically used as model microbial platforms for growth-coupled selection of enzymes and synthetic pathways, non-canonical hosts have increasingly gained attention as alternatives. Among bacterial species, *E. coli* continues to be a preferred option, and the principle of increased fitness over time in the presence of selective pressure has been exploited extensively—epitomized by the classical long-term evolution experiment (LTEE), where cells evolved to optimize carbon utilization pathways towards maximizing growth over 50,000 generations^121^. Building on this notion, and just to mention some key studies over the last 5 years, *E. coli* has been used for the selection and evolution of the activity of several enzymes (e.g., proteases^122^, deaminases^123^ and formate dehydrogenases^80^) and enzymes displaying emergent properties (either natural or engineered, e.g., using non-canonical redox cofactors^124,125^). *S. cerevisiae* has been likewise used to evolve bacterial enzymes, e.g., an efficient tryptophan synthase from *Thermotoga maritima* using OrthoRep^103^.

While these examples illustrate the value of well-established microbial hosts, there are enzymes and pathways involving reaction substrates, intermediates and products that require a more robust host organism for in vivo engineering. Therefore, environmental bacteria thriving in habitats characterized by changing physicochemical conditions, with multiple abiotic and biotic factors (e.g., presence of stressors, salinity levels, pH values and interaction with other microbes) that play a role in shaping their physiology and metabolism, might be suitable hosts for future in vivo engineering projects. *Pseudomonas putida*, a non-pathogenic, Gram-negative soil bacterium^126^, constitutes an archetypal example of a microbe displaying ‘built-in’ robustness, derived from the extreme environments it can colonize. *P. putida* has been used for multiple applications in metabolic engineering, especially towards bioprocesses that require the use of solvents or toxic substrates and products^127^. Although selection schemes based on growth-coupling strategies have been implemented in *P. putida*^128,129^, adopting this bacterium as the host for in vivo evolution of enzymes remains a relatively unexplored endeavor. *P*. *putida* could be an attractive option for the evolution of enzymes generating aromatic aldehydes^130^ and other, similarly reactive intermediates, since such chemical species are part of its native biochemistry, e.g., as metabolites within degradation pathways for aromatic xenobiotics. Moreover, the native metabolic architecture in *P. putida* KT2440 is geared towards catabolic overproduction of reducing power in the form of NADPH^131^, which could further support evolving reactions that require large amounts of redox currency.

Similarly, other strains with properties that are relevant for an automated in vivo engineering process, but not present in *E. coli* or *S. cerevisiae*, could be exploited. The marine bacterium *Vibrio natriegens* is the fastest-growing microbe described so far. A doubling time of less than 10 minutes on rich medium^132^ might enable a faster automated in vivo enzyme engineering process, compared to currently used model species. Since many genetic tools, including plasmids with diverse promoters, ribosome binding sites, and resistance markers^133^, regulatory parts^134^, and a system for multiplex genome editing by natural transformation^135^, are already available for this bacterium, it is likely that it will be harnessed as a chassis for in vivo enzyme engineering in the near future.

Given the expanding wealth of synthetic biology tools available for strain domestication^136–139^, it is not unthinkable that it will become possible to choose any bacterium of interest that is naturally suitable to handle the reaction(s) to be improved or evolved, and use it as a chassis for automated in vivo enzyme engineering. This approach can be extended by using a given enzyme engineering host also directly as a production strain; e.g., the halophilic bacterium *Halomonas bluephagenesis* ^5^ could be used to generate an improved enzyme that is subsequently applied in the high salt medium for the conversion of algal biomass into a desired value-added product. Similarly, use of thermophilic bacteria could be exploited for evolving thermostable enzyme variants^140–142^. Moreover, it might be beneficial to use bacteria that can naturally produce cofactors which are required for an enzyme of interest for the in vivo enzyme engineering procedure. Relevant examples include cofactors such as pyrroloquinoline quinone (PQQ; redox coenzyme in dehydrogenases) or heme (prosthetic group for oxygen-carrying or electron transfer). PQQ is a common coenzyme for alcohol dehydrogenases in *P. putida*^143^ or *Methylobacterium extorquens*^144^; and while the heme biosynthetic pathway is present in *E. coli*^145^, other bacteria, such as the metal-reducing *Shewanella oneidensis*, have many more enzymes that require this prosthetic group^146–148^.

### Towards the automated generation of optimal biocatalysts

The workflow for in vivo-directed evolution of enzymes can be executed through automated setups in a biofoundry. In fact, starting the proposed in vivo enzyme engineering workflow with a specific sensor strain, the task of integrating an efficient module to rescue and enhance cell growth may lead to the requirement of testing different DNA parts in a combinatorial setup. In particular, when the module contains two or more enzymes in a reaction sequence, fine-tuned expression of the underlying genes is required to enable a balanced high carbon flux to maximize growth rate. The latter depends upon the right combinations of multiple DNA parts (i.e., promoter, ribosomal binding site, gene of interest, terminator) in functional transcription units^149^, and the number of strain constructs to be tested can increase rapidly. To meet this challenge with reasonable personnel, material and time expenditure, the standardization, miniaturization and automation of strain engineering workflows is essential.

Emerging biofoundries around the globe are providing automation capabilities for setting up such workflows^19,20,150^ by transferring and combining available methods for modular DNA assembly, highly parallel transformation and incubation, image‐based colony identification and multi‐pin picking, as well as plasmid library preparation using canonical hosts such as *E. coli*^151,152^ or *S. cerevisiae*^153^ as a basis. Recently, robotics-assisted modular cloning^154–158^, high-throughput transformation^159^ and monoclonal colony cultivation and picking^160^ have been introduced for other industrially relevant organisms such as *P. putida* or *Corynebacterium glutamicum*. The correctness of assembled and cloned plasmids can be verified easily with high-throughput using colony PCR or Oxford Nanopore sequencing^161^.

In the next step, the resulting first-generation selection strains will be used for targeted diversity generation with MAGE or other in vivo hypermutators, where the same standardized modules can be integrated into automated workflows. Most importantly, ML methods can be employed to enable autonomous exploration of the enzyme fitness landscape of combinatorial mutagenesis libraries^162^. In the same vein, reliable and autonomous growth phenotyping of resulting second-generation sensor strains has become possible by combining automated microbioreactor platforms^163^ with appropriate data processing tools^164^.

As mentioned above, ALE is another important tool to fully exploit the genetic diversity of sensor strains and enrich the best performing variants. Depending on the required scale, throughput and additional selection pressure^120^, ALE technologies are available for automated operation at laboratory scale^165^, small scale^166^ and single cell level^167^. Genome-wide identification of resulting beneficial mutations or awakened latent enzyme activities is also enhanced by automation of RNASeq technology^168^. Finally, to confirm module activity and identify competing routes that should be inactivated, miniaturized and automated ^13^C-/^15^N-labeling experiments^169^ can be performed in combination with highly informative and accurate LC-QToF mass spectrometry^170^.

**Fig. 5:**
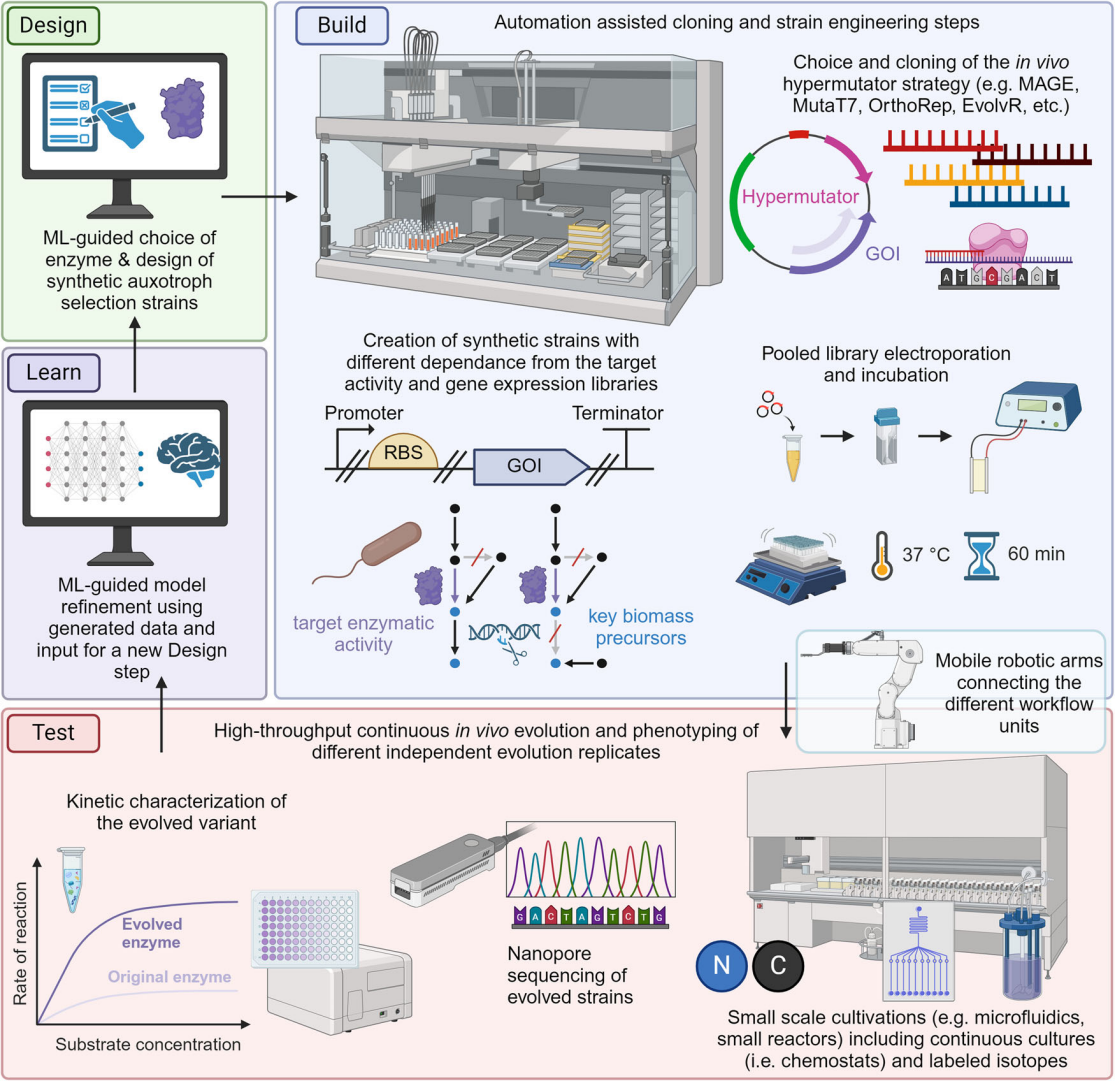
Proposed pipeline for the combined use of machine learning, automation, in vivo mutagenesis, and growth-coupled selection for the directed evolution of enzymes using a biofoundry. Sequential numbers indicate the steps to be taken following the Design-Build-Test-Learn paradigm. ML machine learning, RBS ribosome binding site, GOI gene of interest. For the definition of the hypermutators MAGE, MutaT7, OrthoRep, and EvolvR we refer the reader to the main text. Created with BioRender.com.

The abovementioned technologies can be combined in an automated, ML-guided pipeline. We envision that the combination of in vivo mutagenesis with the screening power of growth-coupled selection will enable to enhance the throughput of biofoundries for enzyme engineering campaigns (Fig. 5). From a technical point of view, the realization of the depicted pipeline is certainly not feasible on the basis of a large solitary platform but requires the combination of a number of customized robot platforms, ultimately connected by mobile robot units, enabling distributed workflows and complex scheduling. Moreover, there are still many pitfalls in integrating specific devices (with different interfaces) into liquid-handling stations, setting up functional and resource-efficient cloning workflows, and implementing a flexible and user-friendly digital infrastructure for running automated experiments, including real-time data processing for loop closure.

Despite these technical challenges, discussions on the perspective of self-driving labs have appeared in the scientific literature^29,30^. Moreover, there is no evidence of their concrete implementation in fully automated experimental setups^28^. We, therefore, expect the possibility of extending such automated workflows also to the in vivo engineering of biocatalysts in the coming future.

### Outlook and final remarks

In this manuscript, we reasoned on the benefits of combining in vivo mutagenesis with growth-coupled selection strategies. As mentioned above, we believe that their use, combined with ML-guided automation, will accelerate enzyme engineering campaigns in the future. However, despite being a promising approach, there are caveats associated with these approaches, which are important to consider and are addressed in this final paragraph.

Relying on growth-coupled selection for in vivo enzyme screening displays an inherent limit in the detection threshold. This is dictated by the minimum enzymatic activity required to replenish the metabolite pool associated with the auxotrophy (i.e., a biomass precursor or a generalist metabolic function). Therefore, if the enzymatic activity is present but at too low level, growth complementation will not occur, and thus, it will not be detected. Therefore, alternative methods can be used, such as transcription factor-based biosensors^171,172^. In principle, if the target enzyme activity can be coupled to transcriptional activation, these types of biosensors could be used as initial step of high-throughput screening and evolution^173^ and detect enzyme activity realizing, e.g., synthesis of a fluorescent protein. Use of such strains might require some adjustments to the automated pipeline presented above, such as the application of FACS to identify and isolate promising candidates. Moreover, the selective pressure imposed on the strains during the in vivo selection can induce the emergence of an underground metabolism or the generation of mutations which bypass the selection. These activities, although scientifically interesting^174,175^, provide a risk of experimental failure, which can be avoided through some measures. These include, i.e., a physiological characterization of the selection strain after its engineering; the use of strains with a quantitatively different dependence on the activity of interest (in terms of mmol essential metabolite g_CDW_^−1^); use of RNAseq or proteomics data to identify possible targets responsible for breaking the selection. Moreover, prior to using the selection strains for growth-coupled experiments, it is recommended to undergo an ALE experiment under selective conditions to identify possible moonlight reactions which can hamper evolutionary campaigns^116^. These pieces of information can then instruct the ML pipeline to curate predictions for gene deletions for the construction of new selection strains.

Besides, some engineering or evolution campaigns might involve enantioselective enzymes or simply enzymes whose activity cannot be coupled to growth. In these cases, the use of growth-coupled selection is not possible, and the approach described in this manuscript would not lead to the attainment of improved biocatalysts.

Another important caveat is related to the use of in vivo hypermutators. Despite all the benefits mentioned above, some techniques display an inherent bias towards a certain type of mutation. This can create diversification in the evolutionary landscape with consequent constrained capacity for long-term sequence space exploration^176^.

Finally, it is important to note that the optimized enzyme obtained at the end of the workflow must be tested in the context of its final purpose. Therefore, other iterative Design-Build-Test-Learn cycles might be required, e.g., in the context of a production strain to assess the effectiveness of the evolution campaign for biomanufacturing, as previously suggested in the use of growth-coupled selection for cell factories optimization^64^.

Box 1 Definitions In vivo engineering: optimization of enzymes or pathways within a living organism. It allows for direct application of in vivo genetic tools to increase enzyme variance and test them within their natural cellular context. When enzymatic activities can be coupled to growth the technique can strongly enhance the testing phase of DBTL cycles and allow selection of improved variants.

DBTL cycle: iterative workflow in bioengineering that involves the design of e.g., enzyme variants, building the according enzyme library, testing functionality of the library, and learning from the results obtained as the foundation for the next iteration. Ideally, this cyclical process allows the development of desired functionalities that can be further optimized by using several cycles.

Selection strains: mutant strains obtained through gene deletions that display an auxotrophy towards one or more essential metabolites (i.e., a biomass precursor) or an impaired general metabolic task (e.g., redox cofactor regeneration).

Hypermutator: synthetic biology tools used to enhance the rate of mutations within a gene of interest. In the context of this manuscript, we refer to in vivo hypermutators to describe tools capable of increasing the mutation rates in the in vivo context of a living organism.

Biofoundry: infrastructure capable of performing molecular biology operations and phenotyping through an automated Design-Build-Test-Learn pipeline. It includes robotic liquid handling, high-throughput analytics systems, and software for the analysis of the large amount of data generated.

Machine learning: a subset of artificial intelligence that self-develops algorithms for, e.g., enzyme engineering by analyzing data from previous experiments for its training. This training enables it to improve performance on a specific task over time without programming, allowing the systems to learn from data and make informed predictions or decisions.
